# Performance of the Reveal Rapid Antibiotic Susceptibility Testing System on Gram-Negative Blood Cultures at a Large Urban Hospital

**DOI:** 10.1128/jcm.00098-22

**Published:** 2022-05-24

**Authors:** Robert Tibbetts, Sheeja George, Reece Burwell, Lara Rajeev, Paul A. Rhodes, Pragya Singh, Linoj Samuel

**Affiliations:** a Henry Ford Health System, Division of Clinical Microbiology, Detroit, Michigan, USA; b Specific Diagnostics, Mountain View, California, USA; Johns Hopkins

**Keywords:** phenotypic antimicrobial susceptibility testing, blood cultures, rapid diagnostics, volatile organic compounds

## Abstract

Timely and effective antibiotic treatment is vital for sepsis, with increasing incidence of antimicrobial-resistant bacteremia driving interest in rapid phenotypic susceptibility testing. To enable the widespread adoption needed to make an impact, antibiotic susceptibility testing (AST) systems need to be accurate, enable rapid intervention, have a broad antimicrobial menu and be easy to use and affordable. We evaluated the Specific Reveal (Specific Diagnostics, San Jose, CA) rapid AST system on positive blood cultures with Gram-negative organisms in a relatively resistant population in a large urban hospital to assess its potential for routine clinical use. One hundred four randomly selected positive blood cultures (Virtuo; bioMérieux) were Gram stained, diluted 1:1,000 in Pluronic water, inoculated into 96-well antibiotic plates, sealed with the Reveal sensor panel, and placed in the Reveal instrument for incubation and reading. The MIC and susceptible/intermediate/resistant category was determined and compared to results from Vitek 2 (bioMérieux) for the 17 antimicrobials available and to Sensititre (Thermo Fisher) for 24 antimicrobials. Performance was also assessed with contrived blood cultures with 33 highly resistant strains. Reveal was in 98.0% essential agreement (EA) and 96.3% categorical agreement (CA) with Sensititre, with just 1.3% very major error (VME) and 97.0%/96.2%/1.3% EA/CA/VME versus Vitek 2. Reveal results for contrived highly resistant strains were equivalent, with EA/CA/VME of 97.7%/95.2%/1.0% with CDC/FDA Antibiotic Resistance Isolate Bank references. Average time to result (TTR) for Reveal was 4.6 h. Sample preparation was relatively low skill and averaged 3 min. We conclude that the Reveal system enables accurate and rapid susceptibility testing of Gram-negative blood cultures.

## INTRODUCTION

Bloodstream infections and associated septic shock are common causes of mortality in hospitals, either as a primary factor or secondary to other disease (1). Current widely used methods for antimicrobial susceptibility testing (AST) require 2 days from blood culture positivity, including at least 1 day to subculture an isolate followed by overnight AST. When the pathogen is resistant to broad-spectrum antimicrobial prophylaxis, this delay can negatively impact patient outcomes (2). Delays in AST results may extend the duration of inappropriate initial antibiotic therapy, leading to both increased mortality and costs associated with sepsis (3). There has been widening interest in rapid AST methods that allow guidance of antimicrobial therapy within an actionable time frame. Rapid AST systems that provide results within an 8-h window could allow for therapeutic interventions in a timely manner (4, 5). Direct-from-blood culture AST testing systems include the commercially available Accelerate Pheno system (Accelerate Diagnostics) (6, 7), rapid disk diffusion (8, 9), and other methods that are currently in development, such as matrix-assisted laser desorption ionization-AST (MALDI-AST) (10). For such an assay to be suitable for broad adoption and routine use, it must be cost-effective, high throughput, and low skill and rapid (minutes) to inoculate, and it should support testing for the ever-increasing menu of relevant antimicrobials. The purpose of the present study was to assess the Reveal AST system (Specific Diagnostics, San Jose, CA) in the context of the above-described criteria.

**FIG 1:**
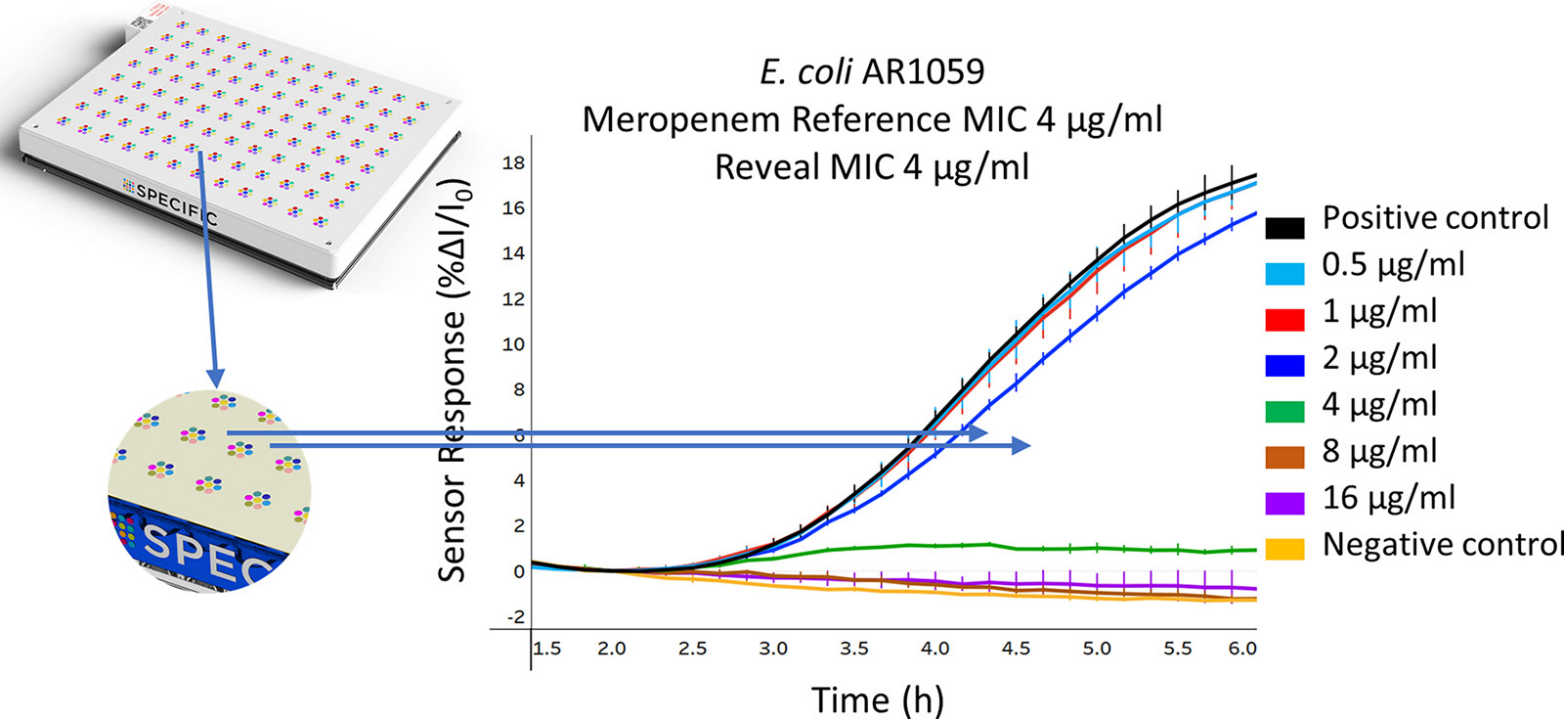
Determination of MIC on the Reveal AST. A sheet of small-molecule sensor arrays is sealed onto a 96-well dried antibiotic plate that is inoculated with the bacterial sample (E. coli AR1095). Above each well is a hexagonal array of 7 sensors that show a colorimetric change in response to the volatile emissions produced by microbial growth. The signals from the positive-control well (black trace) and meropenem wells at concentrations of 2 μg/mL and below indicate growth, while the signals from the negative-control well (yellow trace) and wells with meropenem concentrations above 2 μg/mL are flat, indicating no growth. The divergence of sensor response in growth wells from the negative control allows the determination of MIC, which is 4 μg/mL in this case, matching the CDC broth microdilution result.

The Reveal AST system uses a novel sensor technology to detect the growth of bacterial populations via their emission of volatile organic compounds (VOCs) during growth (11–13), using standard commercially available 96-well dried antibiotic plates (Microscan; Beckman Coulter) (Fig. 1). Here, we present the first clinical evaluation of the Reveal rapid AST system, comparing Reveal antibiograms for 104 randomly selected positive blood cultures with Gram-negative bacteria, along with 33 highly resistant contrived samples from the CDC/FDA Antibiotic Resistance (AR) Isolate Bank against results produced by Sensititre and Vitek 2.

## MATERIALS AND METHODS

### Clinical samples.

Blood culture bottles (BacT/Alert FA and FN PLUS; bioMérieux, Durham, NC) from patients with a suspected bloodstream infection were collected between July 2019 and July 2020 at the primary Henry Ford Health System hospital, a 900-bed facility in Detroit, Michigan, and affiliated community hospitals. Blood culture bottles were incubated in the BacT/Alert VIRTUO system (bioMérieux) until they flagged positive. Bottles were quickly pulled and contents Gram stained (typically within 30 min) on a 24 h per day, 7 days per week (24/7) basis and were then left at room temperature for 0 to 19 h before processing on the Reveal instrument (see Fig. S1 in the supplemental material) (the manufacturer recommends processing within 16 h of bottle positivity). Randomly selected monomicrobial Gram-negative samples were tested for antimicrobial susceptibility using the Reveal system as described below. Samples included the following organisms: Escherichia coli, Klebsiella pneumoniae, Klebsiella oxytoca, Klebsiella aerogenes, Enterobacter cloacae, Citrobacter koseri, and Pseudomonas aeruginosa. Positive blood cultures were also inoculated on blood agar plates to assess purity. Manual CFU counts were performed immediately prior to processing in Reveal by serial 10-fold dilution of the blood culture in saline followed by plating the 10^−6^ dilution on a blood agar plate. All bacterial isolates were stored at −80°C for further analysis.

### Ethical considerations.

The study and its scope, purpose, and methods were all reviewed and approved by the Henry Ford Health System Institutional Review Board.

### Reveal AST assay.

**FIG 2:**
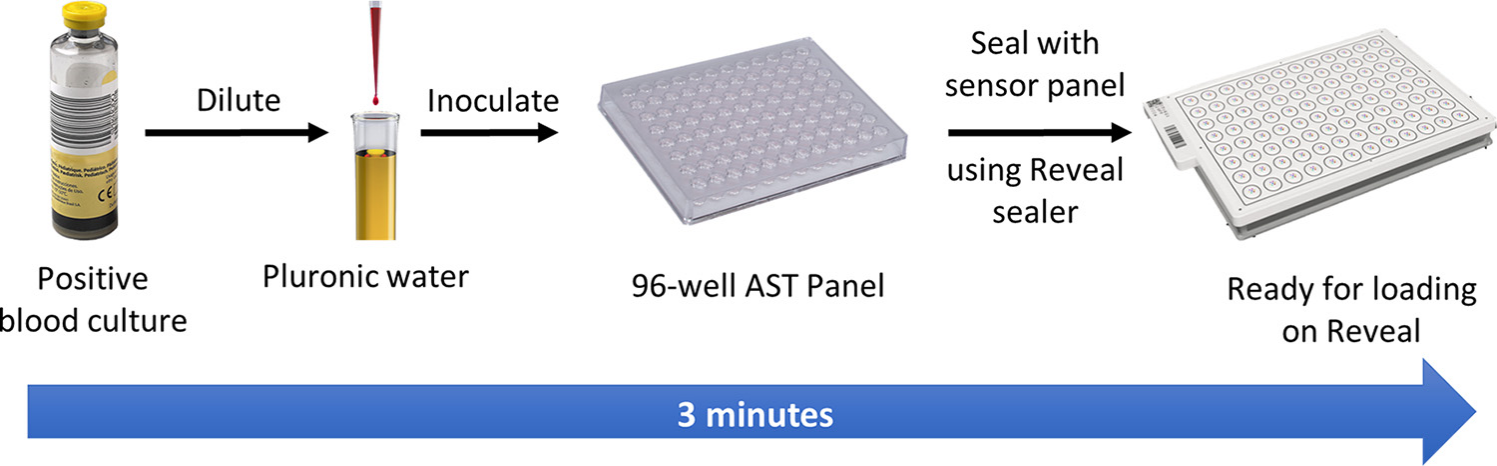
Reveal AST workflow. The workflow for the Reveal AST is a simple ≈3-min process involving inoculation of a single dilution of the positive blood culture into the AST panel, followed by sealing the sensor sheet.

Since the sensors respond to volatiles in the headspace above each well, the inoculum was rapidly prepared by direct dilution of the samples without removal of red blood cells. Positive blood cultures were diluted 1:1,000 in Pluronic water (Beckman Coulter, Brea, CA) and inoculated into Microscan Neg MIC 43 (NM43) dried antibiotic plates (Beckman Coulter) (Fig. 2). In addition to the antimicrobials listed in Table 3, Microscan NM43 plates also contain amoxicillin-clavulanate, cefuroxime, cephalothin, and moxifloxacin, which were excluded from the analysis, since reference data from the comparator methods were not available for these. The plates were covered with the Reveal sensor panel (Specific Diagnostics), which contains arrays of 7 sensors that sit over each well, and sealed using the Reveal plate sealer (Specific Diagnostics). Sample preparation time was approximately 3 min of total time, including syringing and dilution of sample, inoculation, and sealing of the plate (Fig. 2). The sealed plates were barcode-scanned and then placed in the Reveal instrument (Specific Diagnostics), where they were incubated at 37°C and rocked at 100 rpm. As volatiles are emitted from the bacterial population, the sensors respond with a change in color. Each of the 7 sensors in each of the 96 wells is imaged every 10 min to monitor the change in intensity over time. A well with growth media but no antibiotic and another with no growth media acts as a positive growth control and negative no-growth control, respectively (Fig. 1). Pathogen identification (ID), as determined by matrix-assisted laser desorption ionization-time of flight mass spectrometry (MALDI-TOF MS; software version 4.71) (Vitek MS; bioMérieux), on colonies from samples subcultured on solid medium under aerobic conditions at 35°C between 18 and 24 h was entered into the user interface when available. If no ID was obtained using the MALDI, the Vitek 2 using the GN identification cards (software version 9.02) was used. These IDs were combined with Reveal MICs along with Clinical and Laboratory Standards Institute (CLSI) guidelines to produce categorical (S/I/R) results.

### Reference comparator methods.

Reference MICs were obtained with both Vitek 2 (bioMérieux) (GN70 AST card) and Sensititre (Thermo Fisher Scientific, Waltham, MA) (GN4F and ESB1F plates), performed after growth of isolates and preparation of a dilution of 0.5 McFarland in saline (14) according to the manufacturers’ instructions. Sensititre plates were read manually following incubation. Vitek 2 AST cards and Sensititre plates were run from different inocula on different days. MICs and CLSI-interpreted categories (CLSI M100, 25th edition [15]) were compared between the Reveal system and the reference methods. For isolates with elevated cephalosporin MICs (MICs greater than 2 μg/mL for cefotaxime and ceftazidime) that did not have an extended-spectrum beta-lactamase (ESBL) screen test performed by Vitek 2, ESBL testing was performed using a double disk diffusion method, as described by the CLSI, using cefotaxime and ceftazidime alone and in combination with clavulanic acid (15). Species that are known to exhibit intrinsic resistance to included antimicrobials (16) were left out of the analysis. While Reveal yielded results for all strains, Sensititre results could not be obtained for 3 of the 104 strains; therefore, analysis against Sensititre references was performed for 101 strains only (Table 1). Essential agreements (EA) between Reveal and the reference methods were assigned when MIC results from the two methods were within a 2-fold dilution; both on-scale (MICs fall within the concentration range of the panel) and off-scale (MICs that were less than or equal to the lowest concentration or greater than the highest concentration) were included, since the narrow concentration ranges for most drugs on the Microscan panel used in the Reveal system did not allow assessment of evaluable EA for most drugs. Categorical agreement (CA) was determined when susceptible/intermediate/resistant (S/I/R) categorization from Reveal exactly matched that of the reference method; thus, a categorical discrepancy was called when for example the sample was R according to the reference method and I according to Reveal. CA discrepancies were categorized as very major error (VME; falsely susceptible), major error (ME; falsely resistant), and minor error (mE; intermediate susceptibility versus susceptible or resistant) (17).

**TABLE 1:** Overall performance of the Reveal AST system

Parameter or detail	Performance of Reveal AST against:	
	Sensititre	Vitek 2
Parameter, % (no. positive/total no.)		
EA^*c*^	98.0 (2,129/2,173)	97.0 (1,482/1,528)
CA^*c*^	96.3 (2,174/2,258)	96.2 (1,554/1,615)
mE	3.5 (78/2,258)	3.3 (54/1,615)
ME	0.3 (5/1,889)	0.3 (4/1,342)
VME	1.3 (4/313)	1.3 (3/232)
Study set details		
No. of species	7	7
No. of antimicrobials	24	17
No. of strains^*a*^	101	104
No. of total strain-drug pairs^*b*^	2,258	1,615
No. of S strain-drug pairs	1,889	1,342
No. of I strain-drug pairs	56	41
No. of R strain-drug pairs	313	232
% R + I	16.3	16.9

a Sensititre results were not available for 3 strains.

b The total number of strain-drug pairs is the number that was evaluated against Sensititre/Vitek 2 and excludes pairs that show intrinsic resistance (see Table 2 for details).

c ESBL screen results are included in the CA calculations but not the EA.

### Analysis of spiked samples.

In addition to the clinical samples, 33 highly resistant strains from the CDC/FDA Antibiotic Resistance (AR) Isolate Bank were tested from spiked blood cultures on Reveal. The strains tested are listed in Table S1 and represent the following species: A. baumannii (12), C. freundii (4), E. cloacae (8), K. aerogenes (1), K. pneumoniae (4), and P. aeruginosa (4). The strains were subcultured from freezer stocks onto blood agar plates, and a 0.5 McFarland dilution was prepared in sterile saline from pure, isolated colonies and then further diluted 100-fold in sterile saline with 1 mL inoculated into blood culture bottles (BacT/Alert FA Plus; bioMérieux) along with 1 mL of commercially available horse blood (ThermoFisher, Lenexa, KS). Blood culture bottles were incubated in the BacT/Alert VIRTUO system (bioMérieux) until they flagged positive and then were pulled and used for Reveal AST testing as described above. For data analysis, the reference MICs and S/I/R categories provided by the CDC were used as the comparator.

## RESULTS

### Overview.

Two of the 106 (1.9%) positive cultures tested were revealed to be polymicrobial (mixtures of K. pneumoniae with E. coli and Proteus mirabilis with Providencia stuartii) and excluded, leaving 104 samples for which statistics were compiled. Susceptibility results from the Reveal system were compared to both Sensititre and Vitek 2. A total of 2,258 organism-antimicrobial combinations were evaluated, of which 369 (16.3%) were determined to be either resistant or intermediate by Sensititre (Table 1). Reveal AST results were 98.0%/96.3% and 97%/96.2% EA/CA for Sensititre and Vitek 2, respectively, with 1.3%/1.3% VME, 0.3%/0.3% ME, and 3.3%/3.5% mE versus Sensititre and Vitek 2, respectively. Note that these results only pertain to all drugs found on the Sensititre and Vitek 2 panels; additional drugs found on the Microscan panels were not included in the analysis. E. coli was the most common Gram-negative pathogen, being present in ~65% of the samples (Table 2). Other species included in the study were K. pneumoniae (17), P. aeruginosa (11), K. oxytoca (3), E. cloacae (3), K. aerogenes (2), and *C. koseri* (1) (Table 2).

**TABLE 2:** Performance of the Reveal AST system for each species across all antimicrobials

Species and reference	Reveal avg TTR (h)	No. of strains^*a*^	No. of antibiotics^*b*^	No. of strains				% Agreement (no./total no.)		No. of errors		
				Total^*c*^	S	I	R	EA	CA	mE	ME	VME
Sensititre as reference												
*C. koseri*	4.1	1	22	22	22	0	0	100 (22/22)	100 (22/22)	0	0	
E. cloacae	4.5	3	19	57	49	2	6	98.2 (56/57)	94.7 (54/57)	2	0	1
E. coli ^*d*^	4.5	66	24	1,584	1,300	43	241	98.2 (1,491/1518)	96.2 (1,524/1584)	56	3	1
K. aerogenes	4.4	2	19	38	30	2	6	97.4 (37/38)	94.7 (36/38)	1	0	1
K. oxytoca ^*d*^	4.8	3	23	69	69	0	0	95.5 (63/66)	95.7 (66/69)	3	0	
K. pneumoniae ^*d*^	4.6	16	23	368	302	6	60	98.3 (346/352)	96.7 (356/368)	10	1	1
P. aeruginosa	4.6	10	12	120	117	3	0	95.0 (114/120)	94.2 (113/120)	6	1	
Overall		101		2,258	1,889	56	313	98.0 (2,129/2,173)	96.3 (2,174/2,258)	78	5	4
Vitek 2 as reference												
*C. koseri*	4.1	1	14	14	14	0	0	100 (14/14)	100 (14/14)	0	0	
E. cloacae	4.5	3	13	39	33	3	3	97.4 (38/39)	89.7 (35/39)	3	0	1
E. coli ^*d*^	4.5	67	17	1,139	935	19	185	97.6 (1,046/1,072)	97.3 (1,108/1,139)	26	3	2
K. aerogenes	4.4	2	13	26	20	2	4	92.3 (24/26)	84.6 (22/26)	4	0	0
K. oxytoca ^*d*^	4.8	3	16	48	47	1	0	93.3 (42/45)	93.8 (45/48)	3	0	
K. pneumoniae ^*d*^	4.6	17	16	272	218	14	40	95.7 (244/255)	93.8 (255/272)	16	1	0
P. aeruginosa	4.6	11	7	77	75	2	0	96.1 (74/77)	97.4 (75/77)	2	0	
Overall		104		1615	1342	41	232	97.0 (1,482/1,528)	96.2 (1,554/1,615)	54	4	3

a Three strains do not have Sensititre results.

b The number of antibiotics whose results are reported vary by species, because species-drug pairs with intrinsic resistance are excluded from the analysis.

c Total number of AST results evaluated for each species against each comparator method.

d ESBL screen results are not included in EA calculation for E. coli, K. oxytoca, and K. pneumoniae.

To establish that no specific quantification of inoculum was required, CFU counts were obtained for each sample at time of inoculation. As shown in Fig. S2 in the supplemental material, there was no correlation between CFU count and overall AST accuracy or with AST accuracy for specific drugs (Fig. S3), suggesting robustness of the assay to the range of inocula present at bottle positivity. Across all 104 samples, average CFU counts were (1.2 ± 0.6) × 10^9^ CFU/mL. The 33 contrived samples also had a similar range of inoculum at bottle positivity with an average of 3.9 × 10^9^ CFU/mL. Because of the logistics of the 24/7 lab versus the single-shift operation of the study technologist, samples were left on the bench at room temperature for periods ranging from 10 min during normal working hours to up to 19 h for samples pulled and Gram stained after routine working hours (Fig. S1). Five of the samples were run on the Reveal between 16 and 19 h after bottle positivity, outside the manufacturer’s claim of 16 h. The overall EA and CA for these 5 samples were comparable to the rest of the data (Table S2). The accuracy of the Reveal AST was independent of the time that the bottle remained on the bench at room temperature, suggesting that the assay was not impacted by significant delays between bottle pull and assay commencement.

Results for each antimicrobial agent tested on the Reveal AST system are detailed in Table 3, and the results for E. coli against each antibiotic are presented in Table 4 (data for other species are presented in Table S3). Of the 104 strains sampled, 69 were resistant to at least one antimicrobial tested, and 36 were multidrug resistant, as defined by being resistant to at least 1 drug in three or more antimicrobial categories (18). All ESBL-positive strains in the study set were identified by both Sensititre and Vitek 2 tests and the Reveal AST’s ESBL screen test, which uses the MICs from cefotaxime-clavulanate and ceftazidime-clavulanate combination drugs relative to the drug alone (Table 3). One carbapenem-resistant K. aerogenes strain, identified as ertapenem R by both Sensititre and Vitek 2, was called intermediate by the Reveal assay.

**TABLE 3:** Performance of the Reveal AST system by antimicrobial against Sensititre and Vitek 2

Antimicrobial	Reveal avg TTR (h)	Sensititre as reference									Vitek 2 as reference								
		No. of strains^*a*^				% Agreement (no.)		No. of errors			No. of strains^*a*^				% Agreement (no.)		No. of errors		
		Total	S	R	I	EA	CA	mE	ME	VME	Total	S	R	I	EA	CA	mE	ME	VME
Ampicillin	3.5	66	24	42	0	100 (66)	90.9 (60)	6	0	0	67	23	43	1	94.0 (63)	89.6 (60)	7	0	0
Piperacillin	4.6	101	61	23	17	97.0 (98)	93.1 (94)	6	0	1									
Ampicillin/sulbactam	4.6	86	49	16	21	98.8 (85)	66.3 (57)	29	0	0	87	44	31	12	90.8 (79)	81.6 (71)	16	0	0
Piperacillin-tazobactam	4.9	101	97	2	2	96.0 (97)	96.0 (97)	2	0	2	104	97	3	4	94.2 (98)	93.3 (97)	6	0	1
Cefazolin	4.2	86	69	17	0	94.2 (81)	100 (86)	0	0	0	88	68	17	3	97.7 (86)	96.6 (85)	3	0	0
Cefepime	5.2	101	84	17	0	92.1 (93)	100 (101)	0	0	0	104	86	17	1	90.1 (94)	99.0 (103)	1	0	0
Cefotaxime	4.2	91	72	19	0	100 (91)	100 (91)	0	0	0									
Ceftazidime	4.9	101	86	11	4	98.0 (99)	96.0 (97)	3	1	0									
Ceftriaxone	4.1	91	72	19	0	100 (91)	100 (91)	0	0	0	93	74	19	0	97.8 (91)	100 (93)	0	0	0
Cefoxitin	4.1	86	82	2	2	95.3 (82)	88.3 (76)	10	0	0									
Aztreonam	5.1	101	82	19	0	97.0 (98)	99.0 (100)	1	0	0	93	74	19	0	100 (93)	100 (93)	0	0	0
ESBL screen	4.4	85	68	17	0	NA	100 (85)	0	0	0	87	70	17	0	NA	100 (87)	0	0	0
Ertapenem	5.7	91	89	1	1	100 (91)	97.8 (89)	2	0	0	93	90	2	1	97.8 (91)	96.8 (90)	2	0	1
Imipenem	4.9	101	99	0	2	97.0 (98)	97.0 (98)	3	0										
Meropenem	6.0	101	101	0	0	95.0 (96)	95.0 (96)	4	1		104	103	1	0	94.2 (98)	94.2 (98)	4	1	1
Amikacin	4.3	101	101	0	0	100 (101)	100 (101)	0	0		104	104	0	0	100 (104)	100 (104)	0	0	
Gentamicin	4.4	101	95	6	0	100 (101)	99.0 (100)	1	0	0	104	97	6	1	99.0 (103)	100 (104)	0	0	0
Tobramycin	4.5	101	96	5	0	100 (101)	98.0 (99)	2	0	0	104	96	4	4	100 (104)	98 (102)	2	0	0
Ciprofloxacin	3.9	101	80	21	0	98.0 (99)	98.0 (99)	1	1	0	104	81	22	1	98.1 (102)	99.0 (103)	0	1	0
Levofloxacin	4.1	101	80	21	0	98.0 (99)	98.0 (99)	2	0	0									
Tetracycline	4.2	91	67	24	0	98.9 (90)	98.9 (90)	0	1	0									
Tigecycline	4.2	91	91	0	0	100 (91)	100 (91)	0	0		93	93	0	0	100 (93)	100 (93)	0	0	
Nitrofurantoin	4.4	91	81	3	7	98.9 (90)	92.3 (84)	6	0	1	93	77	3	13	98.9 (92)	86.0 (80)	13	0	0
Trimethoprim/sulfa	3.8	91	63	28	0	100 (91)	98.9 (90)	0	1	0	93	65	28	0	97.9 (91)	97.9 (91)	0	2	0

a The number of strains evaluated varies by drug, with species-drug pairs exhibiting intrinsic resistance excluded from the analysis. Three strains do not have Sensititre results. The number of strains evaluated for each comparator was used as the denominator for the respective %EA and %CA calculations.

**TABLE 4:** Reveal AST results for E. coli by antimicrobial against Sensititre (for 66 strains) and Vitek 2 (for 67 strains) reference comparators

Antimicrobial	Avg TTR (h)	Sensititre as reference								Vitek 2 as reference							
		No. of strains			% Agreement (no./66)		No. of errors			No. of strains			% Agreement (no./67)		No. of errors		
		S	I	R	EA	CA	mE	ME	VME	S	I	R	EA	CA	mE	ME	VME
Ampicillin	3.6	24	0	42	100 (66)	90.9 (60)	6	0	0	23	1	43	94.0 (63)	89.6 (60)	7	0	0
Piperacillin	4.4	35	14	17	95.5 (63)	90.9 (60)	5	0	1								
Ampicillin-sulbactam	5.0	35	20	11	100 (66)	62.1 (41)	25	0	0	30	12	25	94.0 (63)	83.6 (56)	11	0	0
Piperacillin-tazobactam	4.5	65	1	0	98.5 (65)	98.5 (65)	1	0		66	0	1	100 (67)	100 (67)	0	0	0
Cefazolin	4.2	54	0	12	93.9 (62)	100 (66)	0	0	0	53	2	12	100 (67)	97.0 (65)	2	0	0
Cefepime	5.2	54	1	11	89.4 (59)	100 (66)	0	0	0	55	1	11	89.6 (59)	100 (67)	0	0	0
Cefotaxime	4.0	54	0	12	100 (66)	100 (66)	0	0	0								
Cefoxitin	4.1	63	2	1	98.5 (65)	90.9 (60)	6	0	0								
Ceftazidime	4.9	58	4	4	100 (66)	95.5 (63)	3	0	0								
Ceftriaxone	4.1	54	0	12	100 (66)	100 (66)	0	0	0	55	0	12	98.5 (66)	100 (67)	0	0	0
Aztreonam	5.3	54	0	12	97.0 (64)	100 (66)	0	0	0	55	0	12	100 (67)	100 (67)	0	0	0
ESBL screen	4.1	54	0	12	NA	100 (66)	0	0	0	55	0	12	NA	100 (67)	0	0	0
Ertapenem	6.2	66	0	0	100 (66)	100 (66)	0	0		66	0	1	98.5 (66)	98.5 (66)	0	0	1
Imipenem	5.0	66	0	0	98.5 (65)	98.5 (65)	1	0									
Meropenem	6.6	66	0	0	92.4 (61)	92,4 (61)	4	1		66	0	1	92.4 (61)	91.0 (61)	4	1	1
Amikacin	4.1	66	0	0	100 (66)	100 (66)	0	0		67	0	0	100 (67)	100 (67)	0	0	
Gentamicin	4.1	60	0	6	100 (66)	100 (66)	0	0	0	61	0	6	100 (67)	100 (67)	0	0	0
Tobramycin	4.5	61	0	5	100 (66)	96.9 (64)	2	0	0	60	3	4	100 (67)	98.5 (66)	1	0	0
Ciprofloxacin	3.8	47	0	19	98.5 (65)	98.5 (65)	0	1	0	47	0	20	97.0 (65)	98.5 (66)	0	1	0
Levofloxacin	3.8	47	0	19	98.5 (65)	98.5 (65)	1	0	0								
Tetracycline	4.0	45	0	21	98.5 (65)	98.5 (65)	0	1	0								
Tigecycline	4.1	66	0	0	100 (66)	100 (66)	0	0		67	0	0	100 (67)	100 (67)	0	0	
Nitrofurantoin	4.2	64	2	0	100 (66)	96.9 (64)	2	0		65	1	1	100 (67)	98.5 (66)	1	0	0
Trimethoprim-sulfa	3.7	42	0	24	100 (66)	100 (66)	0	0	0	44	0	23	98.5 (66)	98.5 (66)	0	1	0

Of a total of 313 resistant antimicrobial-strain assays, just 4 VMEs were observed against the Sensititre and 3 versus Vitek 2. Of these, only 1 (E. cloacae*/*piperacillin-tazobactam) VME was common to both. The 3 other VMEs observed against Sensititre were for K. pneumoniae/piperacillin-tazobactam (an mE against Vitek 2), E. coli/piperacillin (drug not on Vitek 2 AST card), and K. aerogenes/nitrofurantoin (an mE against Vitek 2). The two other VMEs obtained against Vitek 2 were for a single strain of E. coli against ertapenem and meropenem; in this case, Sensititre results were concordant with Reveal’s for both EA and CA, suggesting that Vitek 2 was in error.

Of 1,889 susceptible antimicrobial-strain assay results, Reveal had 3 MEs (also EA errors) in common against both comparator methods, for E. coli/meropenem, K. pneumoniae/trimethoprim-sulfamethoxazole, and E. coli/ciprofloxacin, respectively. Two additional MEs (also EA errors) were observed against Sensititre for drugs not evaluated against Vitek 2: E. coli/tetracycline and P. aeruginosa/ceftazidime. Another ME was obtained for E. coli/trimethoprim-sulfamethoxazole only against Vitek 2; Sensititre was concordant with Reveal’s result for both EA and CA (Table 4 shows E. coli data).

### TTR.

**FIG 3:**
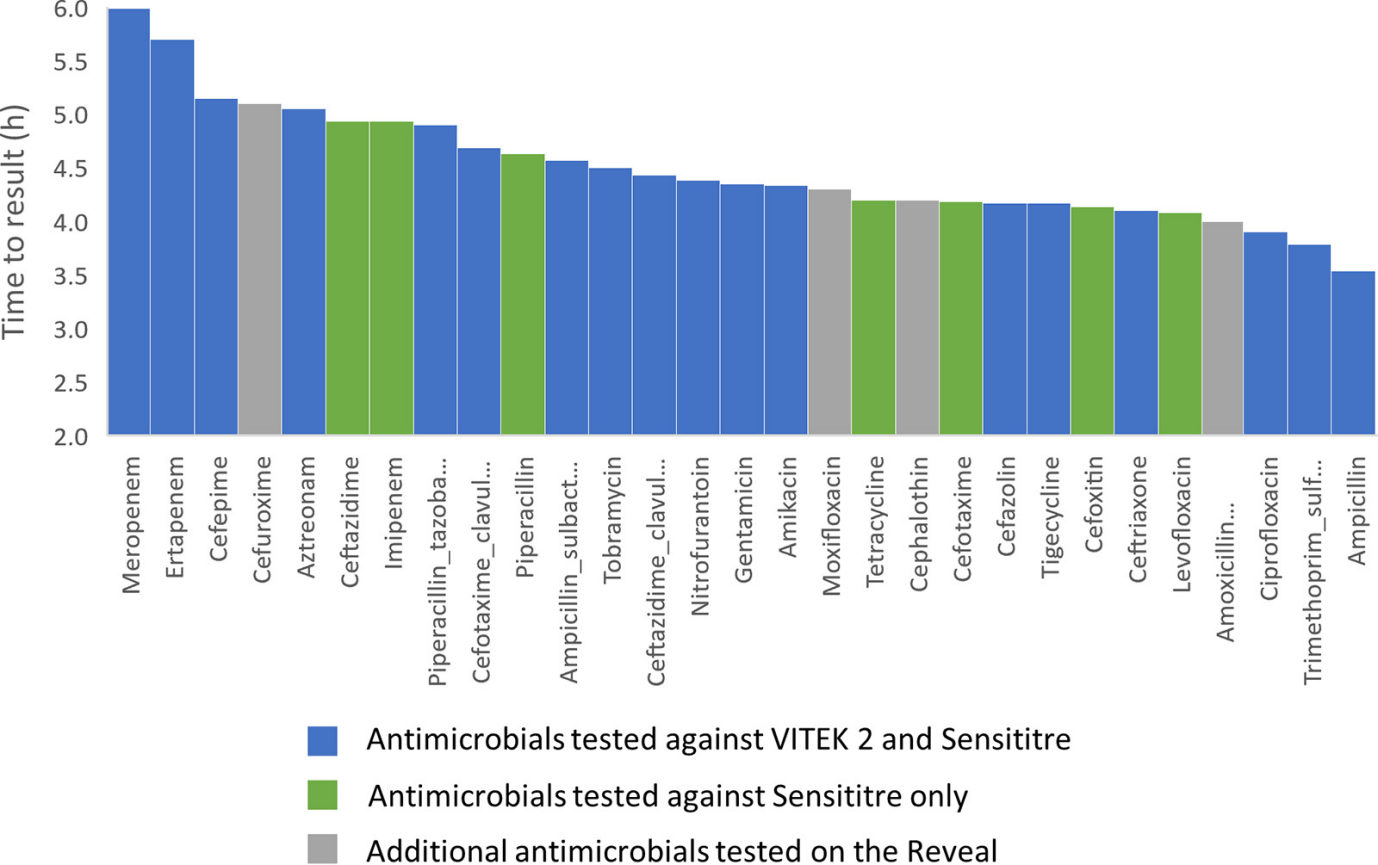
Average time to result for each antimicrobial on Reveal across all species. The results indicated that TTR varied by drug, with meropenem requiring 6 h on average, while ampicillin results were issued in an average of 3.5 h.

The average time to result (TTR) for Reveal was 4.6 h for the clinical samples, ranging from 3.0 h to 6.8 h across all 2,258 strain-drug pairs. The average TTR was similar across the 7 species, from 4.1 h for *C. koseri* to 4.8 h for K. oxytoca (Table 2). The average TTR was drug dependent, ranging from an average of 3.5 h for ampicillin to 6.0 h for meropenem across all species (Table 3 and Fig. 3) or from an average of 3.6 h for ampicillin to 6.6 h for meropenem for E. coli (Table 4). The average TTR for Reveal for the contrived sample set was 4.4 h, ranging from 3.0 h to 8.0 h across all 521 strain/drug pairs.

### Analysis of spiked samples.

To analyze performance of Reveal with more resistant samples, particularly carbapenem-resistant strains, and to test species not well represented in the clinical samples, 33 MDR strains from the CDC/FDA AR repository were tested in contrived samples containing 6 species (A. baumannii, C. freundii, E. cloacae, K. aerogenes, K. pneumoniae, and P. aeruginosa) (Table 5), with 73.7% of the strain-antibiotic pairs tested resistant (Tables 5 and 6), For this data set, Reveal AST had 97.7% EA and 95.2% CA (Table 5) with 1% VME and 0% ME versus the CDC-provided MICs. The 4 VMEs observed were A. baumannii/ampicillin-sulbactam, A. baumannii/tetracycline, E. cloacae/meropenem, and K. aerogenes/piperacillin-tazobactam, respectively.

**TABLE 5:** Performance of Reveal AST with blood samples contrived with CDC AR Bank isolates

Parameter and study detail	Performance with spiked challenge strains
Parameter, % (no. positive/total no.)	
EA	97.7 (509/521)
CA	95.2 (496/521)
mE	4.0 (21/521)
ME	0 (0/122)
VME	1.0 (4/384)
Reveal avg TTR	4.4 h
Study set details	
No. of antibiotics	19
No. of species	6
No. of strains	33
No. of strain/antibiotic pairs	521
No. susceptible	122
No. intermediate	15
No. resistant	384
R (%)	73.7

**TABLE 6:** Reveal AST results for spiked blood cultures by antimicrobial against references provided by the CDC AR Bank

Antibiotic^*a*^	TTR (h)	No. of strains				% Agreement (no.)		No. of errors		
		Total	S	I	R	EA	CA	mE	ME	VME
Ampicillin-sulbactam	4.0	16	0	2	14	93.8 (15)	87.5 (14)	1	0	1
Piperacillin-tazobactam	5.4	33	4	1	28	90.9 (30)	87.9 (29)	3	0	1
Cefepime	4.9	33	6	0	27	100 (33)	100 (33)	0	0	0
Cefotaxime	4.3	29	4	0	25	96.6 (28)	96.6 (28)	1	0	0
Cefoxitin	3.1	4	0	0	4	100 (4)	100 (4)	0	0	0
Ceftazidime	4.2	33	4	1	28	100 (33)	100 (33)	0	0	0
Ceftriaxone	4.1	29	4	0	25	100 (29)	100 (29)	0	0	0
Aztreonam	5.3	21	8	0	13	100 (21)	100 (21)	0	0	0
Ertapenem	4.5	17	5	0	12	94.1 (16)	100 (17)	0	0	0
Imipenem	5.1	33	6	0	27	93.9 (31)	97.0 (32)	1	0	0
Meropenem	5.2	33	6	0	27	97.0 (32)	97.0 (32)	0	0	1
Ciprofloxacin	3.7	33	7	0	26	100 (33)	100 (33)	0	0	0
Levofloxacin	4.0	33	7	1	25	100 (33)	93.9 (31)	2	0	0
Amikacin	4.0	33	14	2	17	100 (33)	93.9 (31)	2	0	0
Gentamicin	4.0	33	9	2	22	100 (33)	93.9 (31)	2	0	0
Tobramycin	3.9	33	10	0	23	100 (33)	93.9 (31)	2	0	0
Tetracycline	4.2	29	9	3	17	93.1 (27)	86.2 (25)	3	0	1
Tigecycline	4.6	17	13	3	1	94.1 (16)	76.5 (13)	4	0	0
Trimethoprim-sulfa	3.6	29	6	0	23	100 (29)	100 (29)	0	0	0

a Only those antibiotics for which the CDC AR Bank provides MICs were used in this analysis.

## DISCUSSION

Here, we report the first clinical evaluation of the Reveal rapid AST system, tested against randomly selected blood cultures with Gram-negative organisms obtained for routine clinical care at a large urban hospital in the Midwest. For the 104 clinical samples, Reveal EA, CA, and VME were 98.0%/96.3%/1.3% and 97.0%/96.2%/1.3% versus Sensititre and Vitek 2, respectively, with an average time to result of 4.6 h on the Reveal. Empiric antimicrobial coverage for Gram-negative bacteremia varies from health system to health system but typically relies on the use of ceftriaxone or cefepime. Our clinical sample data set had 20.4% (19/93) ceftriaxone-resistant and 16.3% (17/104) cefepime-resistant isolates (as determined by the Vitek 2 comparator) (Table 3). Reveal accurately detected 17/17 ESBL-producing clinical isolates (as determined by both Vitek 2 and Sensititre comparators) (Table 3). The results showed comparable performance (98.5% EA, 96% CA, 0.8% VME, 4.3 h TTR) on a set of 33 highly resistant spiked samples (Table 5), suggesting that Reveal performance is generalizable to other populations rather than specific to our patient cohort. For ampicillin-sulbactam, a low CA of 66.3% was observed against Sensititre, although the EA was high (98.8%). This may be attributed to a larger number of strains with MICs at the breakpoints, as seen by a high number of strains in the intermediate category. Interestingly, Reveal showed higher CA of 81.6% for ampicillin-sulbactam against Vitek 2, which also classified fewer strains as intermediate than Sensititre (12 versus 21) (Table 3). The data for E. coli followed a similar pattern for ampicillin-sulbactam, 100% EA with just 62.1% CA against Sensititre (with 20 I strains) and 94% EA/83.6% CA against Vitek 2 (with 12 I strains) (Table 4). Rapid susceptibility results generated by the Reveal could allow for early intervention and reduce duration of inappropriate antimicrobial therapy. In addition, the ability to rapidly detect ESBL and carbapenem resistance has implications for reduction of hospital-acquired infections and infection control.

The Reveal system requires organism ID to be furnished to enable determination of breakpoints, S/I/R interpretation, and selection of therapy. ID can be entered by the user at any time during or after the AST run, after which the appropriate breakpoints are automatically applied by the software, enabling compatibility with workflows not only with multiplexed rapid PCR but also ID methods that require 4 to 5 h of sample preparation for MALDI-TOF-based rapid identification. Thus, with Reveal AST and a rapid ID method, both ID and AST can be obtained within 5 h and as early as 3.5 h following Gram stain results, enabling appropriate therapeutic adjustment at that time (19). A subsequent study in which a rapid direct-from-blood culture ID method is utilized will be required to quantify the time from availability of positive blood culture to integrated and actionable ID+AST results using Reveal.

### Ease of workflow and use with isolate dilutions.

Due to the current shortage of trained laboratory personnel, any novel platform should require minimal hands-on time and be relatively easy to use with flexibility to process individual samples while also allowing for higher throughput if needed. We observed that the Reveal sample preparation process took approximately 3 min of hands-on time on average and required no more skill than inoculation of the disposables of widely used AST systems. Reveal is a modular system, with each 17-inch-wide module processing 4 samples per shift. A stack of 3 modules would allow for 12 samples/shift that could be loaded and unloaded by bench staff. In addition, results on the Reveal for positive blood cultures were not impacted by concentration of organism even when blood culture bottles were left at room temperature for up to 19 h (Fig. S2).

### The availability of rapid TTR.

The average time-to-result (TTR) for the Reveal AST assay was 4.6 h for our data set across all 24 antimicrobials for which comparison was available on Sensititre. The TTR for spiked multidrug-resistant samples was consistent with this finding, averaging 4.3 h. Thus, both accuracy and TTR for a set of multidrug-resistant samples were very similar to that obtained from the clinical samples, suggesting that the accuracies and TTRs reported here are similar regardless of the degree of resistance in the clinical cohort. This TTR potentially allows a positive blood culture sample pulled and Gram stained with actionable phenotypic susceptibility information to be brought to the attention of the provider/pharmacist far more rapidly than current methods. This has the potential to impact patient outcomes and antimicrobial stewardship (20, 21). For institutions that are unable to staff laboratories 24/7, this technology has the potential to mitigate the impact of delayed processing of cultures by reducing delays in obtaining AST.

Other clinically desirable aspects of AST technology include the ability to provide actual MICs and test directly from patient specimens versus bacterial colonies; however, nonclinical attributes such as cost, the amount and size of disposables, and the ability to test multiple specimens simultaneously also need to be taken into consideration. The most widely used emerging technology is the Accelerate Pheno system, and while MICs can be obtained within 7 h direct from positive blood culture bottles, this system, as well as the LifeScale system (Affinity BioSensors, Santa Barbara, CA), can only perform AST on a single specimen per instrument, thereby impeding workflow without having multiple instruments (6, 7). Further, the use of next-generation sequencing methods does not currently provide actual MICs.

There are numerous future technologies being developed for rapid AST, including nuclear magnetic resonance and infrared spectroscopy, flow cytometry, isothermal microcalorimetry, and the use of Raman scattering (SERS) spectra. While these technologies promise to be rapid, some as low as 30 min for a result, many do not allow for testing directly from patient specimens or do not provide actual MICs. For a full and concise review of these technologies, see reference 4.

### Limitations.

There were a number of limitations to this study that will need to be addressed in subsequent work. The study only included Gram-negative isolates in blood cultures, and further study is required to determine performance with Gram-positive organisms. We note that while over 60% of the clinical samples tested were from E. coli, samples from *C. koseri*, K. aerogenes, E. cloacae, and K. oxytoca were too few (1, 2, 3, and 3, respectively) to allow significant conclusions for these species. In addition, our lab does not confirm the presence of inducible AmpC enzymes, and therefore we were unable to assess their presence based on antimicrobial MICs. Furthermore, the CDC AR Bank isolates we used for the spiking studies do not include specific organisms with inducible AmpC enzymes. We also had few resistant isolates for drugs such as piperacillin-tazobactam and nitrofurantoin, making it difficult to interpret the significance of the VMEs obtained for these drugs without additional data. While the antibiotic plate used with Reveal in this study had a broad antimicrobial menu with 29 distinct Gram-negative antimicrobials, certain antibiotics of importance, such as colistin or the newer carbapenem/carbapenemase inhibitor combinations, were not included at this time. Further studies are ongoing to determine assay performance with additional antimicrobials. Because we chose samples at random, there were 2 species (Citrobacter freundii and A. baumannii) on Reveal’s menu that were not represented in the 104 samples that are presented here (although these species were included in the spiked set). Since the Reveal system is essentially a means of observing growth via volatile emission detection, it is reasonable to speculate that this method will be broadly applicable to other species besides those tested here. Further studies are required to evaluate performance with a range of species. We also note that the Reveal AST results are not valid for use with polymicrobial cultures. However, only ~2% of the samples in this study were polymicrobial on subculture, and the results were excluded from the analysis.

In conclusion, we find the Reveal AST assay to be simple to use, with short hands-on time, and for the range of Gram-negative isolates tested it provided susceptibility results with accuracy at a level comparable to the current standard of care. The 4.6-h average time to result when paired with an equally rapid ID result clearly raises the possibility of early de-escalation when paired with an effective information propagation and decision-making system.
