# Supplementary material for: Performance of the Reveal Rapid Antibiotic Susceptibility Testing System on Gram-Negative Blood Cultures at a Large Urban Hospital
Source: J Clin Microbiol. 2022 May 24;60(6):e00098-22. doi: 10.1128/jcm.00098-22 (PMC9199398; doi:10.1128/jcm.00098-22)
Supplement: Supplemental file 1 — Fig. S1 to S3 and Tables S1 to S3. Download jcm.00098-22-s0001.pdf, PDF file, 0.6 MB [file jcm.00098-22-s0001.pdf]

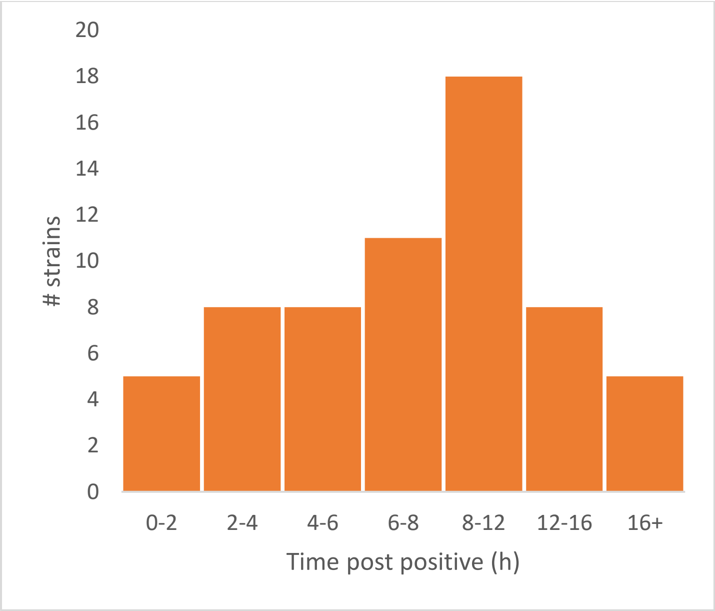

Figure S1. The elapsed time between bottle positivity and loading of the Reveal ranged 0 to 19 hrs. Data collected for 63 strains. 5 strains were run 16+ hours after bottle positivity, of which 4 were run between 16-17 h and 1 was run between 18-19 h.

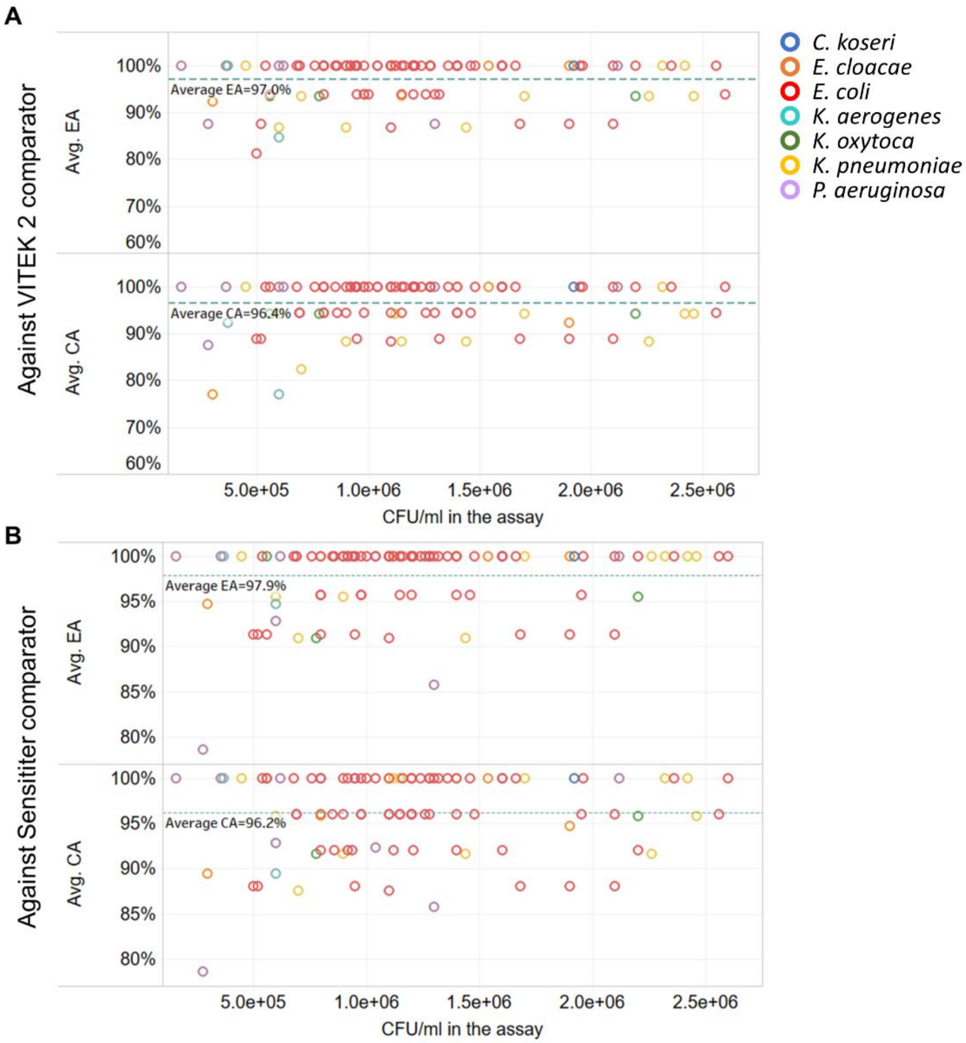

Figure S2. **Reveal results show no correlation between CFU counts and AST accuracy**. Plots of average EA and average CA for each strain across all drugs determined against VITEK 2 (**A**) and Sensititre (**B**) against the CFU/ml in the Reveal assay. The CFU/ml in the assay was a 1000-fold dilution of the CFU/ml determined for the bottle.

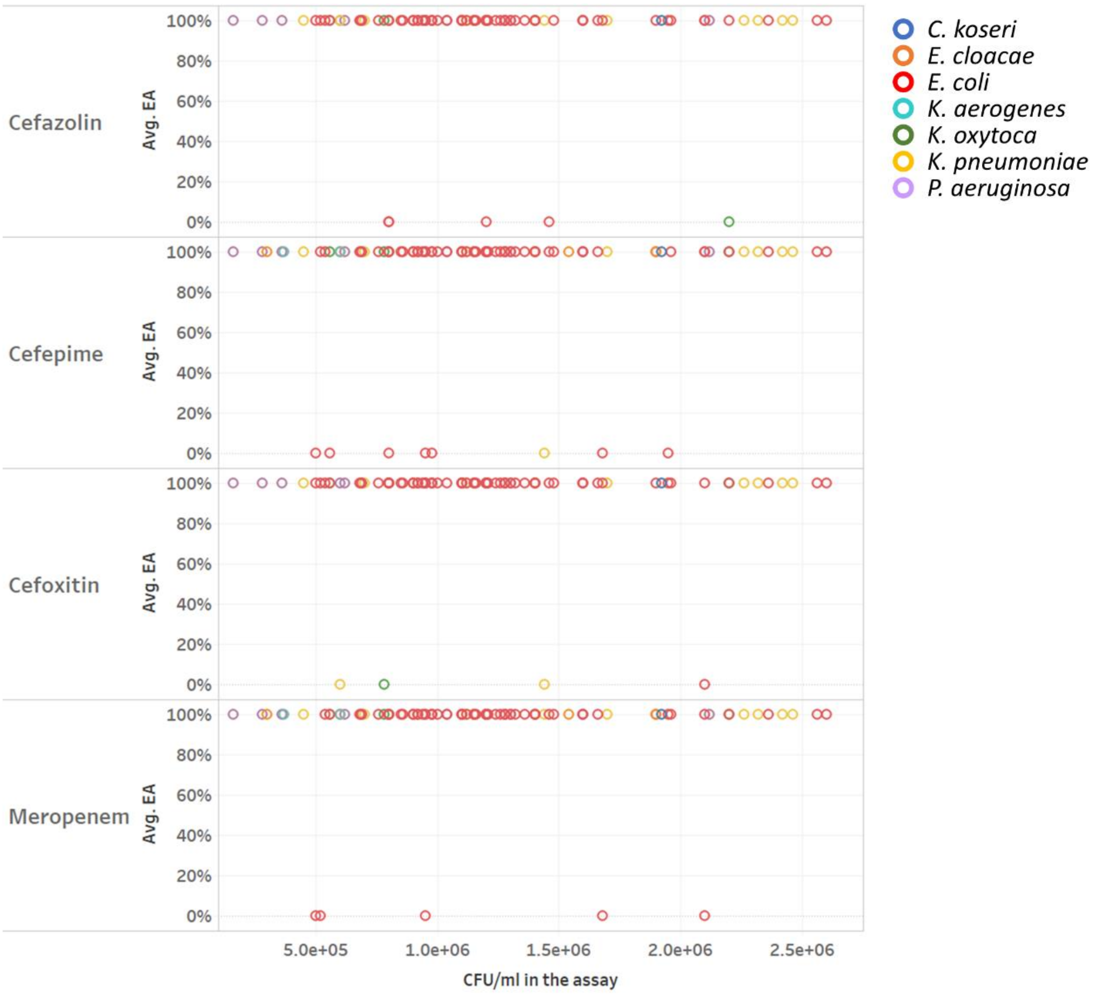

Figure S3. There is no correlation between EA and CFU/ml for any specific drug. The four drugs shown here are those that have four or more EA errors across all clinical samples. Samples that had EA of 0 varied in CFU counts. The CFU/ml in the assay was a 1000-fold dilution of the CFU/ml determined for the bottle.

Table S1. List of CDC AR Bank isolates used for spiked sample analysis

Species	AR bank #	Panel
C. freundii	0021	Enterobacterales carbapenem breakpoint
C. freundii	0022	Enterobacterales carbapenem breakpoint
C. freundii	0023	Enterobacterales carbapenem breakpoint
A. baumannii	0033	Gram-negative carbapenemase detection
A. baumannii	0036	Gram-negative carbapenemase detection
K. pneumoniae	0040	Gram-negative carbapenemase detection
A. baumannii	0045	Gram-negative carbapenemase detection
K. pneumoniae	0049	Gram-negative carbapenemase detection
E. cloacae	0060	Gram-negative carbapenemase detection
A. baumannii	0083	Gram-negative carbapenemase detection
A. baumannii	0101	Gram-negative carbapenemase detection
P. aeruginosa	0108	Gram-negative carbapenemase detection
P. aeruginosa	0110	Gram-negative carbapenemase detection
P. aeruginosa	0111	Gram-negative carbapenemase detection
C. freundii	0157	Enterobacterales carbapenemase diversity
E. cloacae complex	0164	Enterobacterales carbapenemase diversity
P. aeruginosa	0230	*Pseudomonas aeruginosa*
A. baumannii	0275	*Acinetobacter baumannii*
A. baumannii	0278	*Acinetobacter baumannii*
A. baumannii	0279	*Acinetobacter baumannii*
A. baumannii	0280	*Acinetobacter baumannii*
A. baumannii	0282	*Acinetobacter baumannii*
A. baumannii	0283	*Acinetobacter baumannii*
A. baumannii	0299	*Acinetobacter baumannii*
E. cloacae	0448	Ceftazidime/avibactam
K. pneumoniae	0506	Imipenem/relebactam
K. pneumoniae	0507	Imipenem/relebactam
K. aerogenes	0547	Aminoglycoside/tetracycline resistance

E. cloacae	0641	Delafloxacin
E. cloacae	0841	Plazomicin
E. cloacae	0858	Plazomicin
E. cloacae	1042	Meropenem/vaborbactam verification
E. cloacae	1053	Meropenem/vaborbactam verification

Table S2. Reveal performance comparisons for samples run at 0-16 h of bottle positivity and those run after 16 h of bottle positivity.

	TPP 0-16		TPP 16+	
	**Sensititre**	**VITEK 2**	**Sensititre**	**VITEK 2**
# Strains	99	96	5	5
% Essential agreement (#)	98.1% (2041/2081)	97.1% (1424/1467)	95.7% (88/92)	95.1% (58/61)
% Categorical agreement (#)	96.3% (2083/2163)	96.3% (1493/1551)	92.9% (88/95)	95.5% (61/64)
% mE (#)	3.3% (72/2163)	3.4% (52/1551)	6.1% (6/95)	3.0% (2/64)
% ME (#)	0.2% (4/1801)	0.2% (3/1271)	1.2% (1/88)	1.8% (1/58)
%VME (#)	1.3% (4/309)	1.3% (3/228)	0% (0/4)	0% (0/4)

Table S3. Reveal performance by species-drug for all clinical samples other than *E. coli*

	Sensititre as reference								VITEK2 as reference							
**Antimicrobial**	**#S**	**#R**	**#I**	**% EA (#)**	**% CA (#)**	**# mE**	**# ME**	**# VME**	**#S**	**#R**	**#I**	**% EA (#)**	**% CA (#)**	**# mE**	**# ME**	**# VME**
C. koseri (n=1)																
Amikacin	1	0	0	100 (1)	100 (1)	0	0	0	1	0	0	100 (1)	100 (1)	0	0	0
Ampicillin/sulbactam	1	0	0	100 (1)	100 (1)	0	0	0	-	-	-	-	-	-	-	-
Aztreonam	1	0	0	100 (1)	100 (1)	0	0	0	1	0	0	100 (1)	100 (1)	0	0	0
Cefazolin	1	0	0	100 (1)	100 (1)	0	0	0	1	0	0	100 (1)	100 (1)	0	0	0
Cefepime	1	0	0	100 (1)	100 (1)	0	0	0	1	0	0	100 (1)	100 (1)	0	0	0
Cefotaxime	1	0	0	100 (1)	100 (1)	0	0	0	-	-	-	-	-	-	-	-
Cefoxitin	1	0	0	100 (1)	100 (1)	0	0	0	-	-	-	-	-	-	-	-
Ceftazidime	1	0	0	100 (1)	100 (1)	0	0	0	-	-	-	-	-	-	-	-
Ceftriaxone	1	0	0	100 (1)	100 (1)	0	0	0	1	0	0	100 (1)	100 (1)	0	0	0
Ciprofloxacin	1	0	0	100 (1)	100 (1)	0	0	0	1	0	0	100 (1)	100 (1)	0	0	0
Ertapenem	1	0	0	100 (1)	100 (1)	0	0	0	1	0	0	100 (1)	100 (1)	0	0	0
Gentamicin	1	0	0	100 (1)	100 (1)	0	0	0	1	0	0	100 (1)	100 (1)	0	0	0
Imipenem	1	0	0	100 (1)	100 (1)	0	0	0	-	-	-	-	-	-	-	-

Antimicrobial	Sensititre as reference								VITEK2 as reference							
	#S	#R	#I	% EA (#)	% CA (#)	# mE	# ME	# VME	#S	#R	#I	% EA (#)	% CA (#)	# mE	# ME	# VME
Levofloxacin	1	0	0	100 (1)	100 (1)	0	0	0	-	-	-	-	-	-	-	-
Meropenem	1	0	0	100 (1)	100 (1)	0	0	0	1	0	0	100 (1)	100 (1)	0	0	0
Nitrofurantoin	1	0	0	100 (1)	100 (1)	0	0	0	1	0	0	100 (1)	100 (1)	0	0	0
Piperacillin	1	0	0	100 (1)	100 (1)	0	0	0	-	-	-	-	-	-	-	-
Piperacillin/tazobactam	1	0	0	100 (1)	100 (1)	0	0	0	1	0	0	100 (1)	100 (1)	0	0	0
Tetracycline	1	0	0	100 (1)	100 (1)	0	0	0	-	-	-	-	-	-	-	-
Tigecycline	1	0	0	100 (1)	100 (1)	0	0	0	1	0	0	100 (1)	100 (1)	0	0	0
Tobramycin	1	0	0	100 (1)	100 (1)	0	0	0	1	0	0	100 (1)	100 (1)	0	0	0
Trimethoprim/sulfa	1	0	0	100 (1)	100 (1)	0	0	0	1	0	0	100 (1)	100 (1)	0	0	0
E. cloacae (n=3)																
Amikacin	3	0	0	100 (3)	100 (3)	0	0	0	3	0	0	100 (3)	100 (3)	0	0	0
Aztreonam	2	1	0	100 (3)	100 (3)	0	0	0	0	1	2	100 (3)	100 (3)	0	0	0
Cefepime	3	0	0	100 (3)	100 (3)	0	0	0	0	0	0	100 (3)	66.7 (2)	1	0	0
Cefotaxime	2	1	0	100 (3)	100 (3)	0	0	0	-	-	-	-	-	-	-	-
Ceftazidime	2	1	0	100 (3)	100 (3)	0	0	0	-	-	-	-	-	-	-	-
Ceftriaxone	2	1	0	100 (3)	100 (3)	0	0	0	0	1	2	100 (3)	100 (3)	0	0	0
Ciprofloxacin	3	0	0	100 (3)	100 (3)	0	0	0	3	0	0	100 (3)	100 (3)	0	0	0
Ertapenem	2	0	1	100 (3)	66.7 (2)	1	0	0	2	0	1	100 (3)	66.7 (2)	1	0	0
Gentamicin	3	0	0	100 (3)	100 (3)	0	0	0	3	0	0	100 (3)	100 (3)	0	0	0
Imipenem	3	0	0	100 (3)	100 (3)	0	0	0	-	-	-	-	-	-	-	-
Levofloxacin	3	0	0	100 (3)	100 (3)	0	0	0	-	-	-	-	-	-	-	-
Meropenem	3	0	0	100 (3)	100 (3)	0	0	0	2	0	1	100 (3)	100 (3)	0	0	0
Nitrofurantoin	2	0	1	100 (3)	66.7 (2)	1	0	0	2	0	1	100 (3)	66.7 (2)	1	0	0
Piperacillin	2	1	0	100 (3)	100 (3)	0	0	0	-	-	-	-	-	-	-	-
Piperacillin/tazobactam	2	1	0	66.7 (2)	66.7 (2)	0	0	1	2	1	0	66.7 (2)	66.7 (2)	0	0	1
Tetracycline	3	0	0	100 (3)	100 (3)	0	0	0	-	-	-	-	-	-	-	-
Tigecycline	3	0	0	100 (3)	100 (3)	0	0	0	3	0	0	100 (3)	100 (3)	0	0	0
Tobramycin	3	0	0	100 (3)	100 (3)	0	0	0	3	0	0	100 (3)	100 (3)	0	0	0

Antimicrobial	Sensititre as reference								VITEK2 as reference							
	#S	#R	#I	% EA (#)	% CA (#)	# mE	# ME	# VME	#S	#R	#I	% EA (#)	% CA (#)	# mE	# ME	# VME
Trimethoprim/sulfa	3	0	0	100 (3)	100 (3)	0	0	0	3	0	0	100 (3)	100 (3)	0	0	0
K. aerogenes (n=2)																
Amikacin	2	0	0	100 (2)	100 (2)	0	0	0	2	0	0	100 (2)	100 (2)	0	0	0
Aztreonam	1	1	0	100 (2)	100 (2)	0	0	0	0	1	1	100 (2)	100 (2)	0	0	0
Cefepime	2	0	0	100 (2)	100 (2)	0	0	0	0	0	2	100 (2)	100 (2)	0	0	0
Cefotaxime	1	1	0	100 (2)	100 (2)	0	0	0	-	-	-	-	-	-	-	-
Ceftazidime	1	1	0	100 (2)	100 (2)	0	0	0	-	-	-	-	-	-	-	-
Ceftriaxone	1	1	0	100 (2)	100 (2)	0	0	0	0	1	1	100 (2)	100 (2)	0	0	0
Ciprofloxacin	2	0	0	100 (2)	100 (2)	0	0	0	2	0	0	100 (2)	100 (2)	0	0	0
Ertapenem	1	1	0	100 (2)	50 (1)	1	0	0	1	1	0	50 (1)	50 (1)	1	0	0
Gentamicin	2	0	0	100 (2)	100 (2)	0	0	0	2	0	0	100 (2)	100 (2)	0	0	0
Imipenem	2	0	0	100 (2)	100 (2)	0	0	0	-	-	-	-	-	-	-	-
Levofloxacin	2	0	0	100 (2)	100 (2)	0	0	0	-	-	-	-	-	-	-	-
Meropenem	2	0	0	100 (2)	100 (2)	0	0	0	1	0	1	100 (2)	100 (2)	0	0	0
Nitrofurantoin	1	1	0	50 (1)	50 (1)	0	0	1	0	0	2	100 (2)	0 (0)	2	0	0
Piperacillin	1	0	1	100 (2)	100 (2)	0	0	0	-	-	-	-	-	-	-	-
Piperacillin/tazobactam	1	0	1	100 (2)	100 (2)	0	0	0	1	1	0	50 (1)	50 (1)	1	0	0
Tetracycline	2	0	0	100 (2)	100 (2)	0	0	0	-	-	-	-	-	-	-	-
Tigecycline	2	0	0	100 (2)	100 (2)	0	0	0	2	0	0	100 (2)	100 (2)	0	0	0
Tobramycin	2	0	0	100 (2)	100 (2)	0	0	0	2	0	0	100 (2)	100 (2)	0	0	0
Trimethoprim/sulfa	2	0	0	100 (2)	100 (2)	0	0	0	2	0	0	100 (2)	100 (2)	0	0	0
K. oxytoca (n=3)																
Amikacin	3	0	0	100 (3)	100 (3)	0	0	0	3	0	0	100 (3)	100 (3)	0	0	0
Ampicillin/sulbactam	3	0	0	66.7 (2)	33.3 (1)	2	0	0	3	0	0	66.7 (2)	33.3 (1)	2	0	0
Aztreonam	3	0	0	100 (3)	100 (3)	0	0	0	3	0	0	100 (3)	100 (3)	0	0	0
Cefazolin	3	0	0	66.7 (2)	100 (3)	0	0	0	2	0	1	33.3 (1)	66.7 (2)	1	0	0
Cefepime	3	0	0	100 (3)	100 (3)	0	0	0	3	0	0	100 (3)	100 (3)	0	0	0
Cefotaxime	3	0	0	100 (3)	100 (3)	0	0	0	-	-	-	-	-	-	-	-

Antimicrobial	Sensititre as reference								VITEK2 as reference							
	#S	#R	#I	% EA (#)	% CA (#)	# mE	# ME	# VME	#S	#R	#I	% EA (#)	% CA (#)	# mE	# ME	# VME
Cefoxitin	3	0	0	66.7 (2)	66.7 (2)	1	0	0	-	-	-	-	-	-	-	-
Ceftazidime	3	0	0	100 (3)	100 (3)	0	0	0	-	-	-	-	-	-	-	-
Ceftriaxone	3	0	0	100 (3)	100 (3)	0	0	0	3	0	0	100 (3)	100 (3)	0	0	0
Ciprofloxacin	3	0	0	100 (3)	100 (3)	0	0	0	3	0	0	100 (3)	100 (3)	0	0	0
ESBL screen	3	0	0	100 (3)	100 (3)	0	0	0	3	0	0	100 (3)	100 (3)	0	0	0
Ertapenem	3	0	0	100 (3)	100 (3)	0	0	0	3	0	0	100 (3)	100 (3)	0	0	0
Gentamicin	3	0	0	100 (3)	100 (3)	0	0	0	3	0	0	100 (3)	100 (3)	0	0	0
Imipenem	3	0	0	100 (3)	100 (3)	0	0	0	-	-	-	-	-	-	-	-
Levofloxacin	3	0	0	100 (3)	100 (3)	0	0	0	-	-	-	-	-	-	-	-
Meropenem	3	0	0	100 (3)	100 (3)	0	0	0	3	0	0	100 (3)	100 (3)	0	0	0
Nitrofurantoin	3	0	0	100 (3)	100 (3)	0	0	0	3	0	0	100 (3)	100 (3)	0	0	0
Piperacillin	3	0	0	100 (3)	100 (3)	0	0	0	-	-	-	-	-	-	-	-
Piperacillin/tazobactam	3	0	0	100 (3)	100 (3)	0	0	0	2	0	1	100 (3)	100 (3)	0	0	0
Tetracycline	3	0	0	100 (3)	100 (3)	0	0	0	-	-	-	-	-	-	-	-
Tigecycline	3	0	0	100 (3)	100 (3)	0	0	0	3	0	0	100 (3)	100 (3)	0	0	0
Tobramycin	3	0	0	100 (3)	100 (3)	0	0	0	3	0	0	100 (3)	100 (3)	0	0	0
Trimethoprim/sulfa	3	0	0	100 (3)	100 (3)	0	0	0	3	0	0	100 (3)	100 (3)	0	0	0
K. pneumoniae (n=16 for Sensititre; 17 for VITEK2)																
Amikacin	16	0	0	100 (16)	100 (16)	0	0	0	17	0	0	100 (17)	100 (17)	0	0	0
Ampicillin/sulbactam	10	5	1	100 (16)	87.5 (14)	2	0	0	11	6	0	82.3 (14)	82.3 (14)	3	0	0
Aztreonam	11	5	0	100 (16)	100 (16)	0	0	0	12	5	0	100 (17)	100 (17)	0	0	0
Cefazolin	11	5	0	100 (16)	100 (16)	0	0	0	12	5	0	100 (17)	100 (17)	0	0	0
Cefepime	11	5	0	93.8 (15)	100 (16)	0	0	0	12	5	0	88.2 (15)	100 (17)	0	0	0
Cefotaxime	11	5	0	100 (16)	100 (16)	0	0	0	-	-	-	-	-	-	-	-
Cefoxitin	15	1	0	87.5 (14)	81.3 (13)	3	0	0	-	-	-	-	-	-	-	-
Ceftazidime	11	5	0	100 (16)	100 (16)	0	0	0	-	-	-	-	-	-	-	-
Ceftriaxone	11	5	0	100 (16)	100 (16)	0	0	0	12	5	0	94.1 (16)	100 (17)	0	0	0
Ciprofloxacin	14	2	0	93.8 (15)	93.8 (15)	1	0	0	14	2	1	100 (17)	100 (17)	0	0	0

Antimicrobial	Sensititre as reference								VITEK2 as reference							
	#S	#R	#I	% EA (#)	% CA (#)	# mE	# ME	# VME	#S	#R	#I	% EA (#)	% CA (#)	# mE	# ME	# VME
ESBL screen	11	5	0	N/A	100 (16)	0	0	0	12	5	0	N/A	100 (17)	0	0	0
Ertapenem	16	0	0	100 (16)	100 (16)	0	0	0	17	0	0	100 (17)	100 (17)	0	0	0
Gentamicin	16	0	0	100 (16)	100 (16)	0	0	0	17	0	0	94.1 (16)	100 (17)	0	0	0
Imipenem	16	0	0	100 (16)	100 (16)	0	0	0	-	-	-	-	-	-	-	-
Levofloxacin	14	2	0	93.8 (15)	93.8 (15)	1	0	0	-	-	-	-	-	-	-	-
Meropenem	16	0	0	100 (16)	100 (16)	0	0	0	17	0	0	100 (17)	100 (17)	0	0	0
Nitrofurantoin	10	2	4	100 (16)	81.3 (13)	3	0	0	6	2	11	94.1 (16)	47.1 (8)	9	0	0
Piperacillin	10	5	1	100 (16)	100 (16)	0	0	0	-	-	-	-	-	-	-	-
Piperacillin/tazobactam	15	1	0	93.8 (15)	93.8 (15)	0	0	1	14	0	3	88.2 (15)	82.4 (14)	3	0	0
Tetracycline	13	3	0	100 (16)	100 (16)	0	0	0	-	-	-	-	-	-	-	-
Tigecycline	16	0	0	100 (16)	100 (16)	0	0	0	17	0	0	100 (17)	100 (17)	0	0	0
Tobramycin	16	0	0	100 (16)	100 (16)	0	0	0	16	0	1	100 (17)	94.1 (16)	1	0	0
Trimethoprim/sulfa	12	4	0	100 (16)	93.8 (15)	0	1	0	12	5	0	94.1 (16)	94.1 (16)	0	1	0
P. aeruginosa (n=10 for sensititre; 11 for Vitek2)																
Amikacin	10	0	0	100 (10)	100 (10)	0	0	0	11	0	0	100 (10)	100 (10)	0	0	0
Aztreonam	9	0	1	90 (9)	90 (9)	1	0	0	-	-	-	-	-	-	-	-
Cefazolin	0	10	0	100 (10)	100 (10)	0	0	0	0	11	0	100 (10)	100 (10)	0	0	0
Cefepime	10	0	0	90 (9)	100 (10)	0	0	0	11	0	0	90.9 (10)	100 (10)	0	0	0
Cefoxitin	0	8	2	100 (10)	100 (10)	0	0	0	-	-	-	-	-	-	-	-
Ceftazidime	9	0	1	80 (8)	90 (9)	0	1	0	-	-	-	-	-	-	-	-
Ciprofloxacin	10	0	0	100 (10)	100 (10)	0	0	0	11	0	0	100 (10)	100 (10)	0	0	0
Gentamicin	10	0	0	100 (10)	90 (9)	1	0	0	10	0	1	100 (10)	100 (10)	0	0	0
Imipenem	8	0	2	80 (8)	80 (8)	2	0	0	-	-	-	-	-	-	-	-
Levofloxacin	10	0	0	100 (10)	100 (10)	0	0	0	-	-	-	-	-	-	-	-
Meropenem	10	0	0	100 (10)	100 (10)	0	0	0	11	0	0	100 (10)	100 (10)	0	0	0
Piperacillin	9	0	1	100 (10)	90 (9)	1	0	0	-	-	-	-	-	-	-	-
Piperacillin/tazobactam	8	0	2	90 (9)	90 (9)	1	0	0	10	0	1	81.8 (9)	81.8 (9)	2	0	0
Tobramycin	10	0	0	100 (10)	100 (10)	0	0	0	11	0	0	100 (10)	100 (10)	0	0	0
